# A novel Bayesian adaptive design incorporating both primary and secondary endpoints for randomized IIB chemoprevention study of women at increased risk for breast cancer

**DOI:** 10.1186/s13063-022-06930-5

**Published:** 2022-12-05

**Authors:** Byron J. Gajewski, Bruce F. Kimler, Devin C. Koestler, Dinesh Pal Mudaranthakam, Kate Young, Carol J. Fabian

**Affiliations:** 1 Department of Biostatistics & Data Science, The University of Kansas Medical Center, Mail Stop 1026, 3901 Rainbow Blvd, Kansas City, KS 66160 USA; 2 The University of Kansas Cancer Center, Kansas City, USA; 3 Department of Radiation Oncology, The University of Kansas Medical Center, Kansas City, KS 66160 USA; 4 Department of Internal Medicine, Division of Medical Oncology, The University of Kansas Medical Center, Kansas City, KS 66160 USA

**Keywords:** Fibroglandular volume, Ki-67, Bayesian adaptive design, Group sequential monitoring, Breast Cancer prevention, Early phase

## Abstract

**Background:**

Our randomized controlled clinical trial will explore the potential of bazedoxifene plus conjugated estrogen to modulate breast tissue-based risk biomarkers as a surrogate for breast cancer risk reduction. This paper investigates the statistical design features of the trial and the rationale for the final choice of its design. Group sequential designs are a popular design approach to allow a trial to stop early for success or futility, potentially saving time and money over a fixed trial design. While Bayesian adaptive designs enjoy the same properties as group sequential designs, they have the added benefit of using prior information as well as inferential interpretation conditional on the data. Whether a frequentist or Bayesian trial, most adaptive designs have interim analyses that allow for early stopping, typically utilizing only the primary endpoint. A drawback to this approach is that the study may not have enough data for adequate comparisons of a single, key secondary endpoint. This can happen, for example, if the secondary endpoint has a smaller effect than the primary endpoint.

**Methods:**

In this paper, we investigate a trial design called two-endpoint adaptive, which stops early only if a criterion is met for primary and secondary endpoints. The approach focuses the final analysis on the primary endpoint but ensures adequate data for the secondary analysis. Our study has two arms with a primary (change in mammographic fibroglandular volume) and secondary endpoint (change in mammary tissue Ki-67).

**Results:**

We present operating characteristics including power, trial duration, and type I error rate and discuss the value and risks of modeling Bayesian group sequential designs with primary and secondary endpoints, comparing against alternative designs. The results indicate that the two-endpoint adaptive design has better operating characteristics than competing designs if one is concerned about having adequate information for a key secondary endpoint.

**Discussion:**

Our approach balances trial speed and the need for information on the single, key secondary endpoint.

## Background

Testing of potential new interventions for cancer risk reduction generally involves initial early phase investigation. Typically, this single arm pilot feasibility study is followed by a phase II B study in which modulation of a risk biomarker is compared between the new intervention and a control group [1]. Although one biomarker is selected as the primary endpoint, there are generally multiple risk biomarkers of interest. Further favorable modulation of multiple markers makes a stronger case for taking a new promising agent into a phase III cancer incidence trial.

A drawback of standard prevention agents for breast cancer risk reduction is that they frequently induce or worsen vasomotor symptoms (hot flashes). Peri- and postmenopausal women with vasomotor symptoms who are also at increased risk for breast cancer development are often reluctant to take standard medications such as tamoxifen [2]. Duavee™ is the combination of the selective estrogen receptor modulator bazedoxifene 20 mg and conjugated estrogen 0.45 mg and is approved by the FDA for relief of vasomotor symptoms and prevention of osteoporosis [3]. Given clinical safety information and pre-clinical data, we reasoned that this combination might be effective in breast cancer risk reduction in addition to providing relief from hot flashes [4, 5]. We performed a single arm pilot trial in peri- and postmenopausal high-risk women with vasomotor symptoms and noted favorable change in several risk biomarkers including a measure of proliferation (Ki-67) in those individuals whose baseline Ki-67 was > 1% in benign breast tissue and fibroglandular volume (a measure of mammographic breast density [6–8]).

Subsequently, we initiated a National Cancer Institute supported multi-site Phase IIB randomized trial of 6 months bazedoxifene (BZA) 20 mg and conjugated estrogen (CE) 0.45 mg vs wait list control (participants do not receive BZA+CE during the study but can receive BZA+CE after the study is over) in peri- and postmenopausal women at increased risk for breast cancer. Based on pilot data and a power calculation, we plan to accrue 120 women and selected change (smaller volume is better) in fibroglandular volume from baseline to 6 months as our primary endpoint. However, it was important that we be able to design the study keeping in mind a tissue-based endpoint (change in Ki-67 from baseline to 6-months) as well. Further mammographic density and Ki-67 are often not correlated variables [9]. This paper considers the statistical design of this trial and its rationale for choosing a novel Bayesian adaptive design that incorporates the primary and a key secondary endpoint. For more clinical details, see the protocol paper [10].

Adaptive designs have become quite popular, particularly in oncology with over 30,000 results for “adaptive designs oncology” in Google Scholar as of August 10, 2022. One type of adaptive design is the *group sequential design/trial* [11] with over 50,000 results returned when the term “group sequential designs oncology” is queried in Google Scholar also on August 10, 2022. A group sequential design specifies interim analyses that occur in stages with prespecified rules for early termination [11].

### Adaptive design illustrative example with a single endpoint

Our trial will have two stages (*K* = 2). The first stage involves 60 participants equally randomized among the 2 arms in a group sequential design with interim monitoring in which early stopping rules can be applied. Let *k* = 1, 2 be the two stages. To illustrate stopping boundaries for a frequentist group sequential design an intervention is considered better if the |*Z*-statistic|>*c*_*k*_, where *c*_*k*_=1.678 $\sqrt{2/k}$. This is known as O’Brien & Fleming test [11] (p.29, Table 2.3, column 4, row 3) and is an illustrative stopping boundary but is not actually used in the study. The trial stops only if the novel intervention performs better than the control; otherwise, the trial is continued to the maximum sample size. This cut-point results in a trial having a two-sided type I error of 10%. The first cut-point is *c*_*1*_ = 2.373, and the second cut-point is *c*_*2*_ = 1.678.

Many of the adaptive trials we have designed are Bayesian [12], evaluated with frequentist properties [13, 14], which have the added benefit of using prior information as well as inferential interpretation conditional on the data. To illustrate a Bayesian trial using a flat prior distribution, the cut-points are set using posterior probabilities (*pp*) of treatment effect to control the type I error rate. In this case, the cut-points become max(*pp,* 1*-pp*)> *pp*_*k*_, where *pp*_*k*_ = Φ(*c*_*k*_), and Φ(.) is the cumulative distribution function for the standard normal distribution. This cut-point also results in a trial having a two-sided type I error of 10%. The first cut-point is *pp*_*1*_ = .9913, and the second cut-point *pp*_*2*_ = .9533. This is one way to determine the cut-points for a Bayesian design (converting frequentist stopping boundaries to Bayesian posterior probabilities), but other methods may be used. For example, the Bayesian cut-point can be adjusted to preserve the desired type I error with informative prior distributions.

### Novel “two-endpoint adaptive design”

A drawback to a group sequential design that uses only the primary endpoint is that the study may not have enough data for adequate estimation of an important secondary endpoint [15], p.199. To address this issue, previous research supplies methodology for two *co-primary* endpoints [11, 16, 17]. Jennison and Turnbull [11] dedicate an entire chapter to designing group sequential trials with several primary co-endpoints and provide three strategies for handling them in alternative designs: (1) rank the importance of the endpoints, (2) treat them as equal importance, or (3) combine the endpoints using a composite score. For strategy (1), a gatekeeping approach can be used to assure the primary endpoint is used in the final success decision rule [18]. For strategy (2), the final success decision rule can require both endpoints to reach statistical significance, in order for the trial to be successful. This can lead to trial inefficiencies because it will be more difficult for both endpoints to be statistically significant [19]. Strategy (3) requires the building and justification of a weighted combination of the two endpoints [12]. These strategies treat the two endpoints explicitly in the final success criteria; however, we have a different goal whereas we only consider a single endpoint for the final success criteria.

Therefore, we take a hybrid approach of (1) and (2). Specifically, we are interested in a trial design that tests success of a single primary endpoint (rank) but with the desire to stop the trial only if the single, key secondary endpoint also has adequate information for the secondary analysis. Past Bayesian approaches to tackling the co-primary endpoint problem involve several strategies that are usually quite flexible. Some examples include a Dirichlet-multinomial model [20] to combine endpoints in a unified approach as well as adding time-to-event with an exponential-inverse gamma model [21]. Bayesians have also tackled the problem with a utility function [22]. A two-stage Bayesian adaptive approach to the problem uses posterior predictive distributions [23].

Because of their flexibility, the primary tool for assessing operating characteristics of Bayesian designs is through simulation. This allows Bayesians to satisfy guidance of properly handling co-endpoints [19, 24]. Ours is a Bayesian approach that focuses the final analysis on the primary endpoint but with a design that allows for the collection of adequate data for the analysis of the secondary endpoint. We present operating characteristics including power, trial duration, and Type I error rate and discuss the value and risks of modeling Bayesian group sequential designs with primary and secondary endpoints. We also compare our proposed method to two other designs.

## Methods

### Design overview: two-endpoint adaptive design

**Fig. 1:**
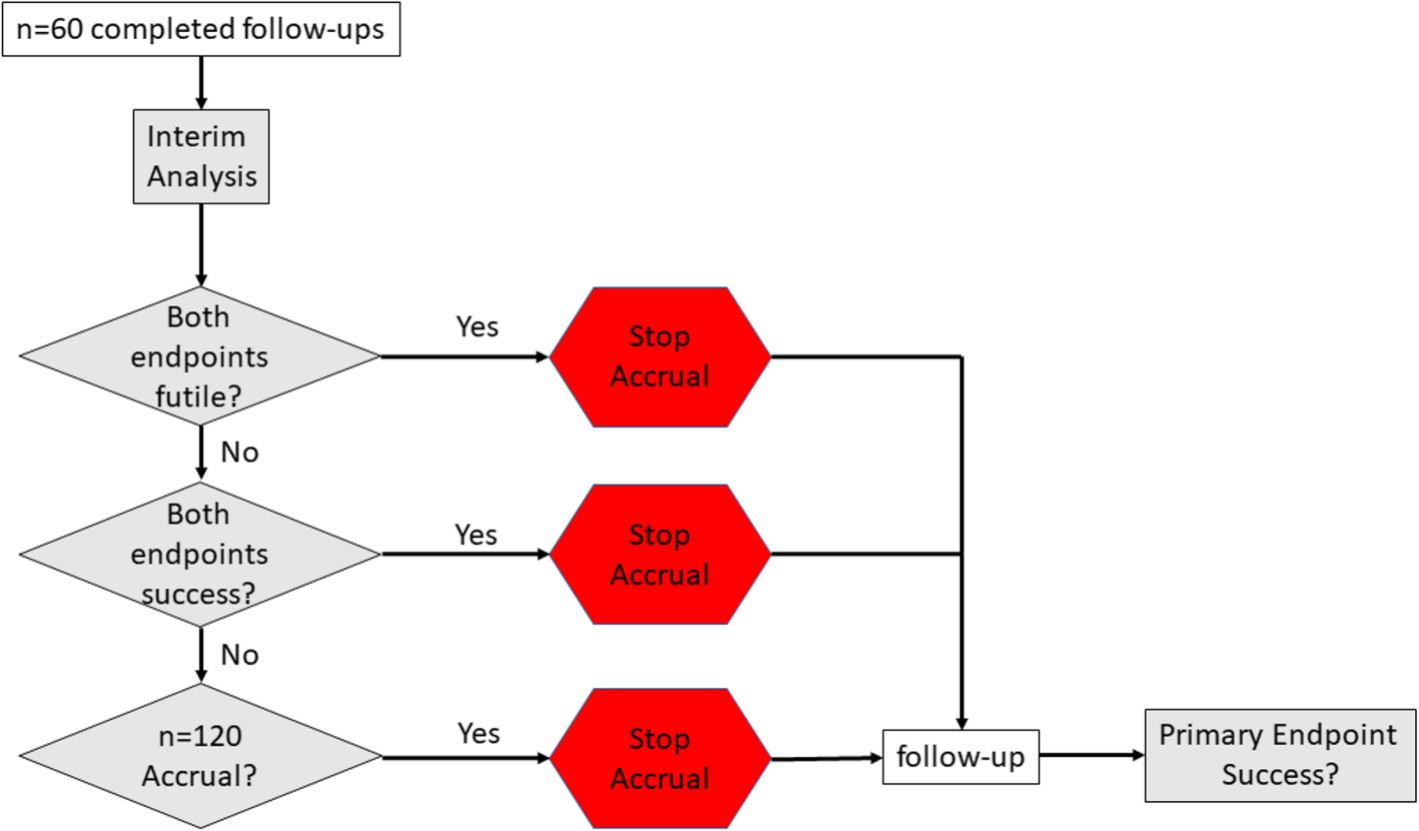
A schematic of the two-endpoint adaptive design. The “*n* = 120 accrual?” asks if we have accrued 120 participants

We provide an overview of our proposed design, called *two-endpoint adaptive design*, with a flow chart in Fig. 1. The posterior cut-points were selected to obtain one-sided type I error rates of 5% and a futility probability of about 25% (under the null hypothesis). We also desired most of the type I error to take place at full accrual. These posterior cut-points were established through simulation and trial and error. Key points of the design are summarized below:

- Minimum sample size: 60;
- Maximum sample size: 120;
- Number of stages: 2;
- Futility stopping rule: posterior probabilities of BZA+CE being better than control in both the primary *and* secondary endpoints are each less than 0.50;
- Success stopping rule: posterior probabilities of BZA+CE being better than control in both the primary *and* secondary endpoints are each greater than 0.9847. We aim to have a one-sided test for success;
- Final success rule: posterior probability of BZA+CE being better than control in the primary endpoint is greater than 0.9517;
- Number of comparative arms: 2;
- Interim analysis: after 60 participants have the opportunity to complete their 6-month visit; and
- Distribution of endpoints: independent normal distributions.

### Statistical model for the two-endpoint adaptive design

Each outcome (change in FGV and change in Ki-67) is independently modeled as a Bayesian two-sample normal distribution using weakly informative priors. The mean drop in FGV for the control arm is *θ*_1, *FGV*_ and for the BZA+CE arm is *θ*_2, *FGV*_. Similar notation is used for Ki-67 for the control and BZA+CE arm, *θ*_1, *Ki* − 67_ and *θ*_2, *Ki* − 67_, respectively. The respective standard deviations for the two endpoints are *σ*_*FGV*_ and *σ*_*Ki* − 67_. The sampling distributions for the two endpoints are, respectively, *Y*_*j*, *FGV*_~*N*(*θ*_*j*, *FGV*_, *σ*_*FGV*_) and *Y*_*j*, *Ki* − 67_~*N*(*θ*_*j*, *Ki* − 67_, *σ*_*Ki* − 67_), where the *j*th intervention is labeled *j* = 1, 2. The prior distributions for the parameters are conjugate for mean and variance parameters and are weakly informative: *θ*_*j*, *FGV*_~*N*(0, 55); *θ*_*j*, *Ki* − 67_~*N*(0, 2); ${\sigma}_{FGV}^2\sim IG\left(\frac{1}{2},\frac{55^2}{2}\right)$; and ${\sigma}_{Ki-67}^2\sim IG\left(\frac{1}{2},\frac{2^2}{2}\right)$. The prior means for each of the endpoints have a mean change of 0 and standard deviation larger than observed in the pilot study. The rationale for setting the prior means to 0 is to say a priori they are the same and letting the trial data dictate posterior differences. The variance parameters have prior estimates larger than the pilot study. These are all weakly informative [25], p. 55 because the prior information is worth only a single prior participant, which is very small compared to the 60 to 120 participants that will be enrolled in the trial.

At each interim, we use the change in FGV and Ki-67 data from all participants who have completed their 6-month visit, denoted in vector form as **Y**_*j,FGV*_ and **Y**_*j,Ki-67*_ respectively and each of length 60 (number of participants to conduct an interim analysis), to calculate the posterior distributions of *θ*_*j*, *FGV*_ and *θ*_*j*, *Ki* − 67_ using Markov Chain Monte Carlo (MCMC). Using the posterior probabilities under each arm, we determine if we should stop the trial early for success or futility. Furthermore, if we have not shown sufficient evidence to stop early, we use the posterior probabilities to continue to the full enrollment of 120 participants.

We stop the trial if the posterior probability that the BZA+CE arm is better than the control for both FGV and Ki-67 endpoints are greater than 0.9847 (success) or if the posterior probability that the BZA+CE arm is better than the control for both FGV and Ki-67 endpoints is less than 0.50 (futility). If the interim analysis does not lead to early stopping, then we continue enrolling participants to 120. If the trial stops early for success or continues to full enrollment, once all the randomized participants have been followed up, we determine trial success if the posterior probability that the BZA+CE arm is better than the control for FGV is greater than 0.9517. The interim success stopping rule and final “strength of evidence” of 0.9517 was chosen to calibrate the one-sided type I error to an acceptable level of no greater than 5%. The interim analysis criteria using both the primary and secondary endpoints were chosen to ensure that enough evidence exists to make meaningful inference on the secondary analysis, which is measured by the expected posterior standard deviation of the difference in the drop of Ki-67, SD(*θ*_2, *Ki* − 67_ − *θ*_1, *Ki* − 67_), SD stands for posterior standard deviation. There is no particular-sized SD of the difference in Ki-67 change that we were targeting, rather to identify the SD that would provide a high posterior probability that the BZA+CE arm is better than the control with respect to Ki-67.

### Simulation scenarios

We use trial simulations to evaluate the proposed design. We consider several possible underlying truths for the mean response, representing the null hypothesis, several alternative hypotheses, and for trial execution, variables such as accrual and dropout. We generate data according to those truths and run through the specified design for each of these scenarios. For comparison, we look at a group sequential design that ignores the secondary outcome in the interim analysis as well as a one-endpoint fixed sample size design (described later). We repeat this process to create multiple “virtual trials” and track the operations of each trial. How the virtual subject-level data is generated is the focus of this section.

#### Virtual subject response for two arms

We label ${\theta}_{j, FGV}^0$ as the true FGV mean change from baseline to 6 months for the *j*th intervention where *j* = 1, 2. The superscript “0” is used to emphasize these are the parameters generating the data, not the parameters for inference after observing data. We also label ${\theta}_{j, Ki-67}^0$ as the true Ki-67 mean change from baseline to 6 months for the *j*th intervention. The respective standard deviations for the two endpoints are ${\sigma}_{FGV}^0$ and ${\sigma}_{Ki-67}^0$. The sampling distributions for the two endpoints are, respectively, ${Y}_{j, FGV}\sim N\left({\theta}_{j, FGV}^0,{\sigma}_{FGV}^0\right)$and ${Y}_{j, Ki-67}\sim N\left({\theta}_{j, Ki-67}^0,{\sigma}_{Ki-67}^0\right)$. We initially focus on four scenarios for treatment arm effects. From a pilot study, the standard deviation parameters for the FGV and Ki-67 respectively are ${\sigma}_{FGV}^0=22.6$ and ${\sigma}_{Ki-67}^0=1.7$. For the first (null scenario, H0), we assume that the true mean responses for FGV are ${\boldsymbol{\uptheta}}_{FGV}^0=\left[2.5,2.5\right],$and the mean responses of Ki-67 are ${\boldsymbol{\uptheta}}_{Ki-67}^0=\left[0.0,0.0\right]$. The alternative scenarios, H1, are all shown in Table 1 and reflect the scenarios of no effect (null), expected, large, and worse (H1).

**Table 1:** Virtual subject response means for change in FGV and Ki-67. The expected effect scenario comes from results from the pilot study [8]. The rest of the effects are defined for calculating type I error (none) as well as reasonable deviations from the expected scenario

	FGV (primary)	FGV (primary)	Ki-67 (secondary)	Ki-67 (secondary)
Effect scenario	Control	BZA+CE	Control	BZA+CE
None (H0)	2.5	2.5	0.0	0.0
Expected (H1)	2.5	− 20.1	0.0	− 0.4
Large (H1)	0.0	− 30.0	0.0	− 1.8
Worse (H1)	0.0	5.0	0.0	0.2

The risk we are trying to manage in the trial design is to balance the use of resources (e.g. trial duration and sample size) while having acceptable probability of making the right decision about the primary endpoint (e.g., success and futility). At the same time, we would like to have sufficient information regarding the single, key secondary outcome for analysis. Therefore, we investigate further scenarios where the FGV effect is large (− 30) but the Ki-67 secondary measure varies in its effect, namely from − .1 to − 1.7. We will also look at this Ki-67 variation when the FGV is very large (− 45).

#### Accrual rate patterns

The timing of interim analysis and the length of trial duration depend on the accrual rate. We assume the weekly accrual (*n*_*t*_) follows a Poisson distribution with parameter *λ* (*n*_*t*_~Poisson(*λ*)). It is assumed that the true average rate of accrual is *λ* = 0.76 participants/week. The accrual rate is based on the target of enrolling 120 participants and having 6-month follow-up within 3.5 years. We have multiple sites to enroll from should the accrual rate be lower than desired.

As a sensitivity analysis we also assumed that it took some time to accelerate accrual, called “slow to 0.76.” During the first 13 weeks the accrual improves linearly from 0 to .25; then, the next 13 weeks improves linearly from 0.25 to .5, and the next 13 weeks improves linearly from .5 to a steady state of 0.76 participants/week.

### Dropout rate

We simulate subjects dropping out of the trial with an overall rate of 10%, which results in missing data for both endpoints. The rationale of the assumption comes from the pilot study that had the same primary endpoint as the current design. As a sensitivity analysis we also assumed a smaller dropout rate of 5%.

### Approach to trial simulation algorithm

Using simulation, we calculate operating characteristics including power, trial duration, and type I error rate and discuss the value and risks of modeling the sequential trials. Repeating 10,000 times, we simulate the number of trial participants recruited and that have completed follow-up (i.e., have data available) for both endpoints at the 6 month visit. In our trial, recruitment will not be halted during the time required for the 60th subject to complete the 6-month timepoint. Thus, the minimum number of subjects will be 60. However, with a shorter interval before evaluation, it would be feasible to suspend enrollment until the interim analysis is completed. The accrual rate determines the time participants are enrolled as well as the time of the interim analysis, that is, when 60 participants have the opportunity to observe their 6-month visit. Each trial could stop early for success/futility or continue to 120 participants, depending on the Bayesian quantities. Then, we repeat the methods outlined above using the different assumptions for the trial parameters, which includes virtual subject responses (Table 1) for the primary and secondary endpoints. The size, duration, and probability of decisions are calculated for each of these assumptions.

We justify the 10,000 simulations performed using a margin of error calculation. The maximum 95% margin of error is $1.96\ \sqrt{.5\ast \left(1-.5\right)/10000}$<0.01. However, under a type I error of 0.05 or power of 0.95, the margin of error is much smaller: $1.96\ \sqrt{.95\ast \left(1-.95\right)/10000}<.004$.

We implemented the simulations in the Fixed and Adaptive Clinical Trials Simulator (FACTS^TM^) [26], which is a Bayesian and frequentist adaptive/fixed design simulation platform. We have also constructed an OpenBUGS program shown in the Appendix.

### Alternative designs

We compare the key operating characteristics for the two-endpoint adaptive design against other designs to highlight the relative strengths and weaknesses in terms of the number of trial participants, trial duration, power, probability of futility, and standard deviation of group differences for the secondary endpoint. First, we perform a *one-endpoint fixed* design using only one endpoint. The one-endpoint fixed design enrolls the full 120 participants with no stopping rules. Trial success occurs if the posterior probability BZA+CE arm is better than the control for the FGV endpoint is greater than 0.95. Second, we have a design that is adaptive but only uses the primary endpoint at the interim analysis to stop for success or futility. Called *one-endpoint adaptive*, stopping criteria for determining success or futility occurs when at least 60 participants are randomized and have had the opportunity to observe their 6-month visit. We stop the trial if the posterior probability BZA+CE arm is better than control for the FGV endpoint is greater than 0.9913 (success) or if the posterior probability BZA+CE arm is better than control for the FGV endpoint is less than 0.25 (futility). If the interim analysis does not lead to early stopping, then we continue enrolling participants to 120. If the trial stops for success early or continues to full enrollment, we determine trial success if the posterior probability that the BZA+CE arm is better than control for the FGV endpoint is greater than 0.9533. All designs here were calibrated to one-sided type I error of 5% by adjusting their stopping rules (i.e. posterior probability an arm has the maximum utility). Both of the adaptive designs were calibrated to have the same futility rate ~25% under the scenario of no effect.

## Results

For the breast cancer prevention trial, we perform simulations based on the scenarios shown in Table 1 and we compare the three designs: one-endpoint fixed, one-endpoint adaptive, and two-endpoint adaptive, on several operating characteristics with results shown in Table 2. These results correspond to accrual rate of 0.76 and dropout rate of 10%.

**Table 2:** Operating characteristics for one-endpoint fixed, one-endpoint adaptive, and two-endpoint adaptive designs. For a range of scenarios, we compare the mean number of subjects enrolled, probability of early and late success, power, probability of early futility, and trial duration. Early and late success probabilities tell us what proportion of the power took place at the interim or final respectively. Mean duration is follow-up time for trials (in weeks) that stopped early for either success or futility

Design	Scenario	Mean subj.	Ppn early success	Ppn late success	Power	Ppn early futility	Mean duration (weeks)
One-endpoint fixed	No effect	120	0.00	0.04	0.04	0.00	184
One-endpoint adaptive	No effect	110	0.01	0.04	0.05	0.24	171
Two-endpoint adaptive	No effect	110	0.00	0.05	0.05	0.25	170
One-endpoint fixed	Expected effect	120	0.00	0.90	0.90	0.00	184
One-endpoint adaptive	Expected effect	106	0.34	0.56	0.90	0.00	165
Two-endpoint adaptive	Expected effect	118	0.04	0.86	0.90	0.00	182
One-endpoint fixed	Large effect	120	0.00	0.99	0.99	0.00	184
One-endpoint adaptive	Large effect	96	0.60	0.39	0.99	0.00	151
Two-endpoint adaptive	Large effect	93	0.66	0.33	0.99	0.00	148
One-endpoint fixed	Worse	120	0.00	0.00	0.00	0.00	184
One-endpoint adaptive	Worse	98	0.00	0.00	0.00	0.55	154
Two-endpoint adaptive	Worse	99	0.00	0.00	0.00	0.53	155

**Fig. 2:**
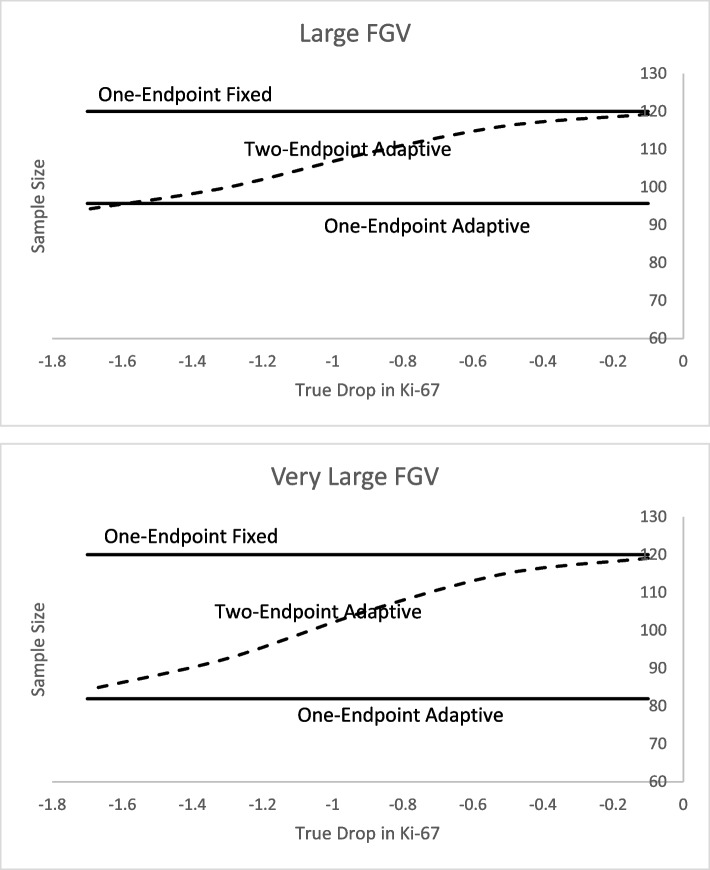
Expected sample size when primary endpoint (FGV) is **a** large (− 30 vs 0) and **b** very large (− 45 vs 0) and the secondary endpoint (Ki-67) varies in its effect

**Fig. 3:**
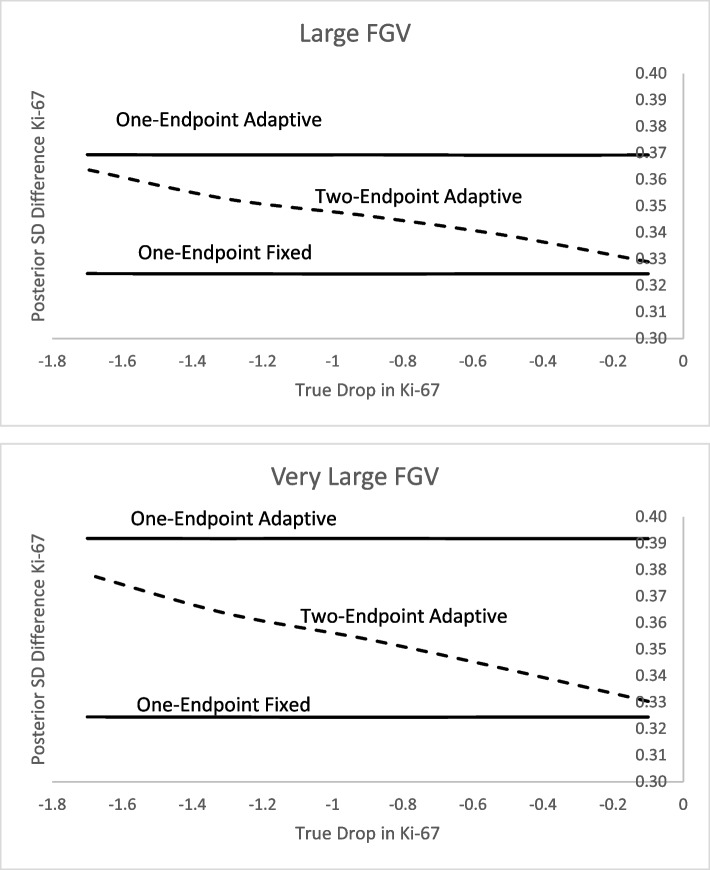
Expected posterior standard deviation when primary endpoint (FGV) is **a** large (− 30 vs 0) or **b** very large (− 45 vs 0) and the secondary endpoint (Ki-67) varies in its effect

Under the no effect scenario, all three designs have type I error rates close to 5%, with a majority of the false positives happening as late successes. We typically calibrate our studies to not be too aggressive in stopping early. For example, under the null hypothesis (no effect), the probability of stopping early is very low whereas continuing to the maximum sample size is high. This is accomplished with a higher posterior probability cut-point for early success relative to late success. The adaptive designs lead to smaller and faster studies than the fixed because of the early futility rule. When assuming expected effect, all designs have about 90% power but two-endpoint adaptive is a bit faster and smaller than the one-endpoint fixed design. The one-endpoint adaptive is markedly smaller and faster than both one-endpoint fixed and two-endpoint adaptive designs. This is an expected result because the one-endpoint fixed does not have an early stopping rule and the two-endpoint adaptive has a more stringent stopping rule as both primary and secondary endpoints need to reach the early stopping cut-point. The one-endpoint adaptive has one early stopping cut-point. For the large effect assumption, the two-endpoint adaptive and one-endpoint adaptive design are approximately the same relative to each other but much smaller and faster than the one-endpoint fixed design because it does not have an early stopping rule. They all are very powerful (99%). The reason the two adaptive designs are about the same is that the effect size is so small, and there is only one interim analysis. If we had more than two interims, then the two-endpoint adaptive would be slower than the one-endpoint adaptive. For the worse scenario, the futility rates lead to faster and smaller trials for the adaptive designs relative to one-endpoint fixed. Using these operating characteristics alone, one would lean towards the one-endpoint adaptive design. However, after examining the expected sample sizes and posterior standard deviation of the difference in Ki-67 scores in Figs. 2 and 3, we see more insight on the relative strengths of two-endpoint adaptive design.

For both large FGV and very large FGV the one-endpoint fixed design maintains a sample size of 120 whereas the one-endpoint adaptive design maintains just under 100 for large FGV and just above 80 for very large FGV. The sample sizes for the two-endpoint adaptive design stay between the other designs but are closer in size to one-endpoint adaptive for large differences in arms for Ki-67 and are closer in size to one-endpoint fixed design for smaller differences in Ki-67. This is when the advantage of the two-endpoint adaptive over the one-endpoint adaptive is evident, because the standard deviation for the difference in secondary endpoint is always smaller than the one-endpoint adaptive. Thus providing more information for the important secondary endpoint (see Figs. 2 and 3). To summarize, the two-endpoint adaptive approach is a compromise between one-endpoint adaptive and one-endpoint fixed. It is smaller in size than the one-endpoint fixed but has a lower standard deviation than the one-endpoint adaptive.

Table 3 compares the two-endpoint adaptive design operating characteristics for sensitivity to the accrual and dropout assumptions. There is very little difference in size, power, and futility rates but the slow to 0.76 participants/week took about 14 to 15 weeks longer for trial duration than when the assumption was a constant 0.76 participants/week.

**Table 3:** Operating characteristics for the two-endpoint adaptive design with a range of scenarios and dropout rates. We compare mean number of subjects enrolled, probability of early and late success, power, probability of early futility, and trial duration (in weeks)

Scenario	Accrual rate	Dropout rate	Mean Subj.	Ppn early success	Ppn late success	Power	Ppn early futility	Mean duration (weeks)
No effect	0.76	0.1	110	0.00	0.05	0.05	0.25	170
No effect	Slow to .76	0.1	110	0.00	0.04	0.04	0.24	184
No effect	0.76	0.05	110	0.00	0.05	0.05	0.25	170
No effect	Slow to .76	0.05	110	0.00	0.05	0.05	0.25	184
Expected effect	0.76	0.1	118	0.04	0.86	0.89	0.00	182
Expected effect	Slow to .76	0.1	118	0.04	0.86	0.90	0.00	195
Expected effect	0.76	0.05	118	0.04	0.87	0.91	0.00	181
Expected effect	Slow to .76	0.05	118	0.04	0.86	0.90	0.00	195
Large effect	0.76	0.1	94	0.66	0.33	0.99	0.00	148
Large effect	Slow to .76	0.1	94	0.66	0.33	0.99	0.00	162
Large effect	0.76	0.05	92	0.69	0.30	0.99	0.00	147
Large effect	Slow to .76	0.05	93	0.68	0.31	0.99	0.00	161
Worse	0.76	0.1	99	0.00	0.00	0.00	0.53	155
Worse	Slow to .76	0.1	99	0.00	0.00	0.00	0.51	170
Worse	0.76	0.05	98	0.00	0.00	0.00	0.54	155
Worse	Slow to .76	0.05	98	0.00	0.00	0.00	0.54	168

## Discussion

Typically, in phase II trials, a primary endpoint is defined for successful decision-making. This can be done with a fixed trial design or a group sequential design (adaptive), with the latter potentially shortening the trial duration using fewer participants. The reliance on only the primary endpoint in adaptive trial decision making results in a trial that regrets not collecting enough information for a single, key secondary endpoint. Some of the literature uses both the primary and secondary endpoints in final success or futility decision-making [23]. However, using both endpoints can lead to trial inefficiencies because one increases the type II error (e.g., lowers the power) [19]. Therefore, we proposed a hybrid approach which uses both primary and secondary endpoints for trial success or futility for interim decision-making, but only the primary endpoint for final analysis. This trial design allows for sufficient information to be collected to maintain good estimation properties for the secondary endpoint. Trial operating characteristics for the proposed design are almost always in between the fixed and adaptive designs that only use the primary endpoint. Our results show that the power is very similar across trials, but the proposed design is more efficient than the fixed design and provides more information for the secondary endpoint than the adaptive with just a primary endpoint.

A limitation of the approach is that it is highly dependent on the discrepancy in the importance of the primary endpoint versus the secondary endpoint. For example, FGV and Ki-67 were rank-ordered as primary versus secondary. In no way is one endpoint drastically clinically more important than the other endpoint; in fact, they are thought to be uncorrelated risks of breast cancer. This relative importance is typical in breast cancer prevention since a primary endpoint, such as mortality, would not be a practical endpoint because it is so rare. However, if mortality was the primary endpoint, it would not be wise to use our design since mortality is much more important than say candidate secondary endpoint Ki-67.

The two-endpoint adaptive design is proposed for a specific breast cancer study with FGV as the primary endpoint and Ki-67 as the secondary endpoint. This design could be applied to other trials where there is a clear primary and important secondary endpoint. For example, weight loss or smoking cessation, where the primary and secondary endpoints could be short- and long-term follow-ups, respectively. There may be scientific reasons for short-term success of a novel therapy, say 1 month, but the investigative team might want more information on long-term success, say 6 months. Using the two-endpoint adaptive design would allow more information to be collected about the long term endpoint.

Interestingly, the two-endpoint adaptive design could be extended to more than one important secondary outcome. Simply adjust the early stopping decision rule for all the endpoints of interest but still only declare trial success for the single primary endpoint. Further, this multiple-endpoint adaptive design strategy can be used for correlated outcomes. One can either adjust the model for correlations or keep the independent assumption but simulate the virtual subject responses to see what adjustments to the operating characteristics occur when the data are correlated. If there is a bias in the design, for example larger than 5% type I error, one can adjust the stopping and/or success decision rules.

## Conclusion

Our proposed design is an adaptive design with a primary endpoint (FGV) and an important secondary endpoint (Ki-67). This two-endpoint adaptive design uses both endpoints for early stopping but only the primary endpoint for final decision making. This approach balances trial speed and the need for information on the secondary endpoint.

## Data Availability

No real-world data was collected in this study. OpenBUGS code to perform model fitting is provided in an Appendix.

## Appendix

### OpenBUGS Code

model

{

for (i in 1:n)

{

y1[i]~dnorm(theta1[Group[i]],invsige12) #FGV likelihood

y2[i]~dnorm(theta2[Group[i]],invsige22) #Ki-67 likelihood

}

for (j in 1:2)## 1 is control, 2 is BZA+CE

{

theta1[j]~dnorm(0,0.0003305785) # FGV mean priors

theta2[j]~dnorm(0,.25) # Ki-67 mean priors

}

invsige12~dgamma(.5,1510) # FGV precision prior

invsige22~dgamma(.5,2) # Ki-67 precision prior

sigma1<-1/sqrt(invsige12)

sigma2<-1/sqrt(invsige22)

Pr1<-step(theta1[1]-theta1[2]) #FGV: probability BZA+CE better than control

Pr2<-step(theta2[1]-theta2[2]) #Ki-67: probability BZA+CE better than control

}
